# Response to “Comment on ‘Optimal Exposure Biomarkers for Nonpersistent Chemicals in Environmental Epidemiology’”

**DOI:** 10.1289/ehp.1611282

**Published:** 2016-04-01

**Authors:** Antonia M. Calafat, Matthew P. Longnecker, Holger M. Koch, Shanna H. Swan, Russ Hauser, Lynn R. Goldman, Bruce P. Lanphear, Ruthann A. Rudel, Stephanie M. Engel, Susan L. Teitelbaum, Robin M. Whyatt, Mary S. Wolff

**Affiliations:** 1 Centers for Disease Control and Prevention, Atlanta, Georgia, USA; 2 National Institute of Environmental Health Sciences, National Institutes of Health, U.S. Department of Health and Human Services, Research Triangle Park, North Carolina, USA; 3 Institute for Prevention and Occupational Medicine of the German Social Accident Insurance, Ruhr-Universität Bochum, Bochum, Germany; 4 Icahn School of Medicine at Mount Sinai, New York, New York, USA; 5 Harvard T.H. Chan School of Public Health, Harvard University, Boston, Massachusetts, USA; 6 Milken Institute School of Public Health, George Washington University, Washington, DC, USA; 7 British Columbia Children’s Hospital, Vancouver, British Columbia, Canada; 8 Silent Spring Institute, Boston, Massachusetts, USA; 9 University of North Carolina at Chapel Hill, Chapel Hill, North Carolina, USA; 10 Mailman School of Public Health, Columbia University, New York, New York, USA

We appreciate the opportunity to respond to the letter from Stahlhut et al. regarding our Brief Communication. We stressed the importance of biospecimen integrity and the potential danger of unrecognized contamination of convenience samples, particularly with ubiquitous environmental chemicals such as bisphenol A (BPA) and phthalates.

We did not discuss the important area of experimental research and specifically pharmacokinetic studies, although we based our argument partly on knowledge of concentration changes in various compartments postexposure. We agree that information from pharmacokinetic models is quite valuable and note that experimental studies that use isotope-labeled materials are not susceptible to extraneous contamination. Such experimental studies do not support using polar metabolites, such as unmetabolized BPA, as biomarkers in epidemiologic studies (Thayer et al. 2015). For example, even in situations that may result in exposures higher than background levels, such as handling cash register receipts, BPA serum concentrations are below or near the detection limit and much lower than urinary concentrations (Thayer et al. 2016).

The figure in our Brief Communication revealed the sharp increase in the number of publications using blood-based polar biomarkers over the past 15 years, especially etiologic studies. Our main point was that urine is the most dependable biomonitoring matrix for population research, a position that Stahlhut et al. also support in their letter.

Target-organ dose may inform biological models, but measuring this dose is not always possible, although it can be inferred from pharmacokinetic studies. For environmental epidemiology, reliable measurements in urine can be used to quantify exposures.

A suitable exposure biomarker involves more than detecting the analyte with precise and accurate methods. For pervasive chemicals and particularly for archived samples, specimen integrity must be confirmed. This is true for any matrix, including urine (Guidry et al. 2015; Koch et al. 2012), to ensure valid results.
